# Supplementary material for: Chromothripsis during telomere crisis is independent of NHEJ, and consistent with a replicative origin
Source: Genome Res. 2019 May;29(5):737–49. doi: 10.1101/gr.240705.118 (PMC6499312; doi:10.1101/gr.240705.118)
Supplement: Supplemental Material [file supp_gr.240705.118_Supplemental_file_1.zip › contigs/annotated_contigs/DB38/contig.2.DB38_length_838_mean_cov_9.69331742243.docx]

**DB38_length_838_mean_cov_9.69331742243**

```
TCTTTAATCCATCTTGAATTGATTTTTGTATAAGGTGTAAGGAAGGGATCCAGTTTCAGCTTTCTACCTGTGGCTAGCCAGTTTTCCCA
 >chr4:19641002-19641305 + E=2e-170 p=3e-02
GCACCATTTATTAAATAGGGAATCCTTTCCCCATTGCTTGTTTTTCTCAGGTTTGTCAAAGATCAGATAGGTGTAGATATGCGACGTTA

TTCCTGAGGGCTCTGTTCCGTTCCATTGATCTATATCTCTGTTTTGGTACCAGTACCATGCTGTTTTGGTTACTGTAGCCTTGTAGTAT

AGTTTGAAGTCAGGTAGTGTGATGCCTCCAGGTT|TG|ACCTCATGATCCACCTGCCTTAGCCTCCCAAAGTGCTGTGATTACAGGTGT
                                   >chr4:19767819-19768362 + E=6e-301
GAGCCATCAAGCCAGGCCTATTTGGAAACTTTTGAGCTTCCTGCATATGTATGTTCATATGTTTCCTAAGAAATAGGGAGTTTTCAGCT

ATCATTTTATTAAATATATTTTATGTGATTTTTTTCCTGTCTCTTCTCTGGAATTTTCATAATGTGAATTTTTATTTACTTAATAGTCT

TCCATAATTCCTGTAGGTTTTCTTCATTCTTTTGGTTTTTCTTTTATTTTTTGGTCTGATTGAGTTATTACTTAAAAATTTGCCTTTAC

ATTCAGAAATCCTATCTTCTGCTTGATGTAGTCTGTTGTTATATATTATGTTTTATTTAATTCATTCAATTTTTCAGCTCCAAGATTTC

TGGGATTTGTTTTGTTTTTGTTTTTGTTTTTGTTTTTGTTTGTTGAGACAGAGTCTCACTCTGTTGCCCAGGCTGGAGTGCAGTGGCAT

CATCTCAGCTCACTGCAGCCTCAACCTCCTGGGCTCAGT
```
